# Supplementary material for: Low-cost, scalable, and automated fluid sampling for fluidics applications
Source: HardwareX. 2021 May 31;10:e00201. doi: 10.1016/j.ohx.2021.e00201 (PMC9123361; doi:10.1016/j.ohx.2021.e00201)
Supplement: Supplementary data 1 [file mmc1.docx]

**Supplementary Figures & Tables**

**Low-cost, scalable, and automated fluid sampling for fluidics applications**

A. Sina Booeshaghi^1,*^, Yeokyoung (Anne) Kil^2,*^, Kyung Hoi (Joseph) Min^3^, Jase Gehring^4^, and Lior Pachter^5,6^

1. Department of Mechanical Engineering, California Institute of Technology, Pasadena, California
2. Department of Medical Engineering, California Institute of Technology, Pasadena, California
3. Department of Electrical Engineering and Computer Science, Massachusetts Institute of Technology, Cambridge, Massachusetts
4. Department of Genome Sciences, University of Washington, Seattle, Washington
5. Division of Biology and Biological Engineering, California Institute of Technology, Pasadena, California
6. Department of Computing and Mathematical Sciences, California Institute of Technology, Pasadena, California

* Authors contributed equally

Address correspondence to [lpachter@caltech.edu](mailto:lpachter@caltech.edu)


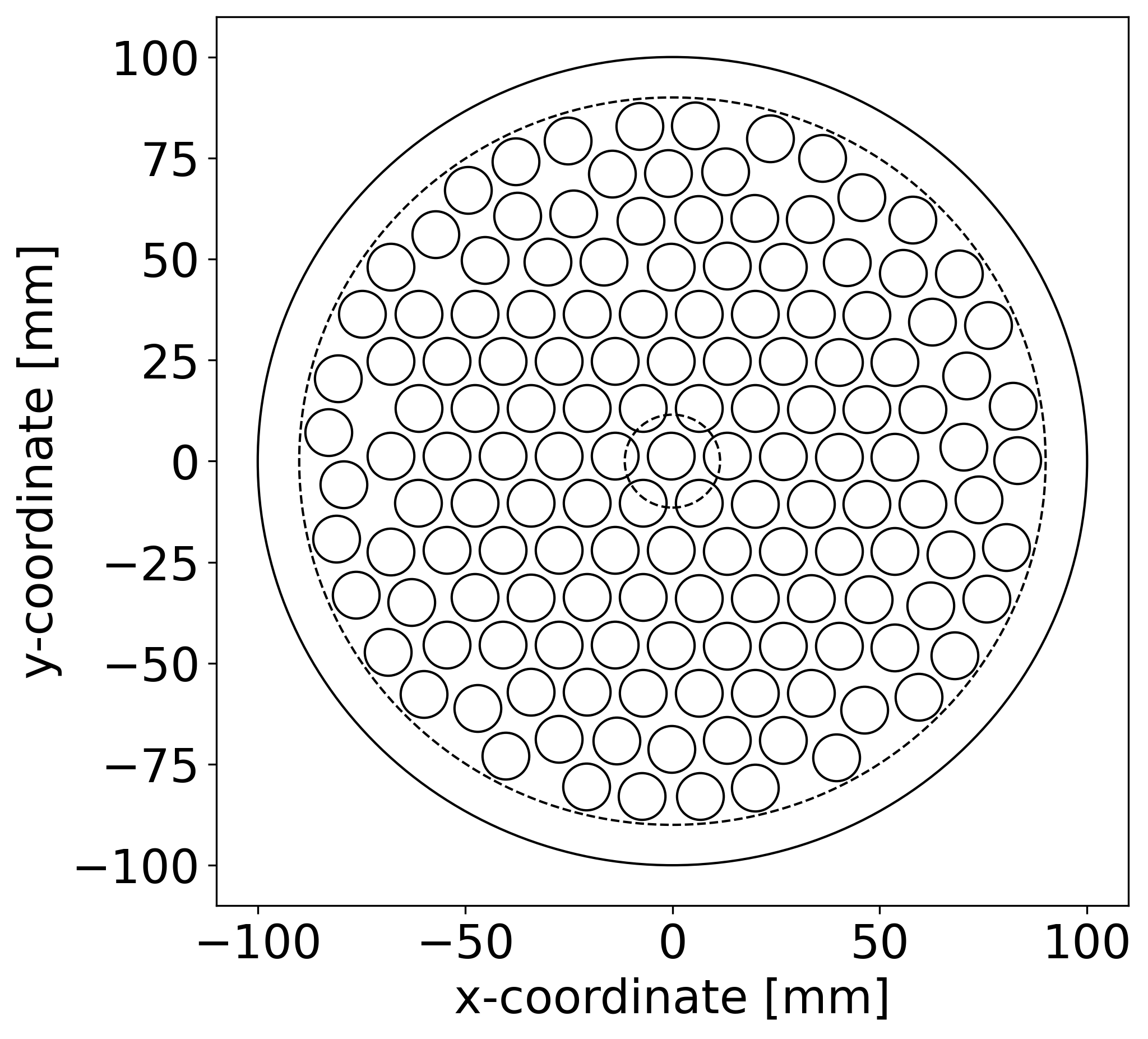


**Supplementary Figure 1:** Optimal packing of disks of diameter 13.5 mm (11 mm tube hole size plus 2.5 margin) in a disk of diameter 180 mm. The solid line corresponds to the outer diameter of the tube rack, the smaller dashed line corresponds to the effective area available for placing tubes, and the smallest dashed line corresponds to the empty area on the colosseum tube rack where no tubes can be placed. [Code (<https://github.com/pachterlab/BKMGP_2021/blob/main/analysis/archimedian_spiral.ipynb>)]


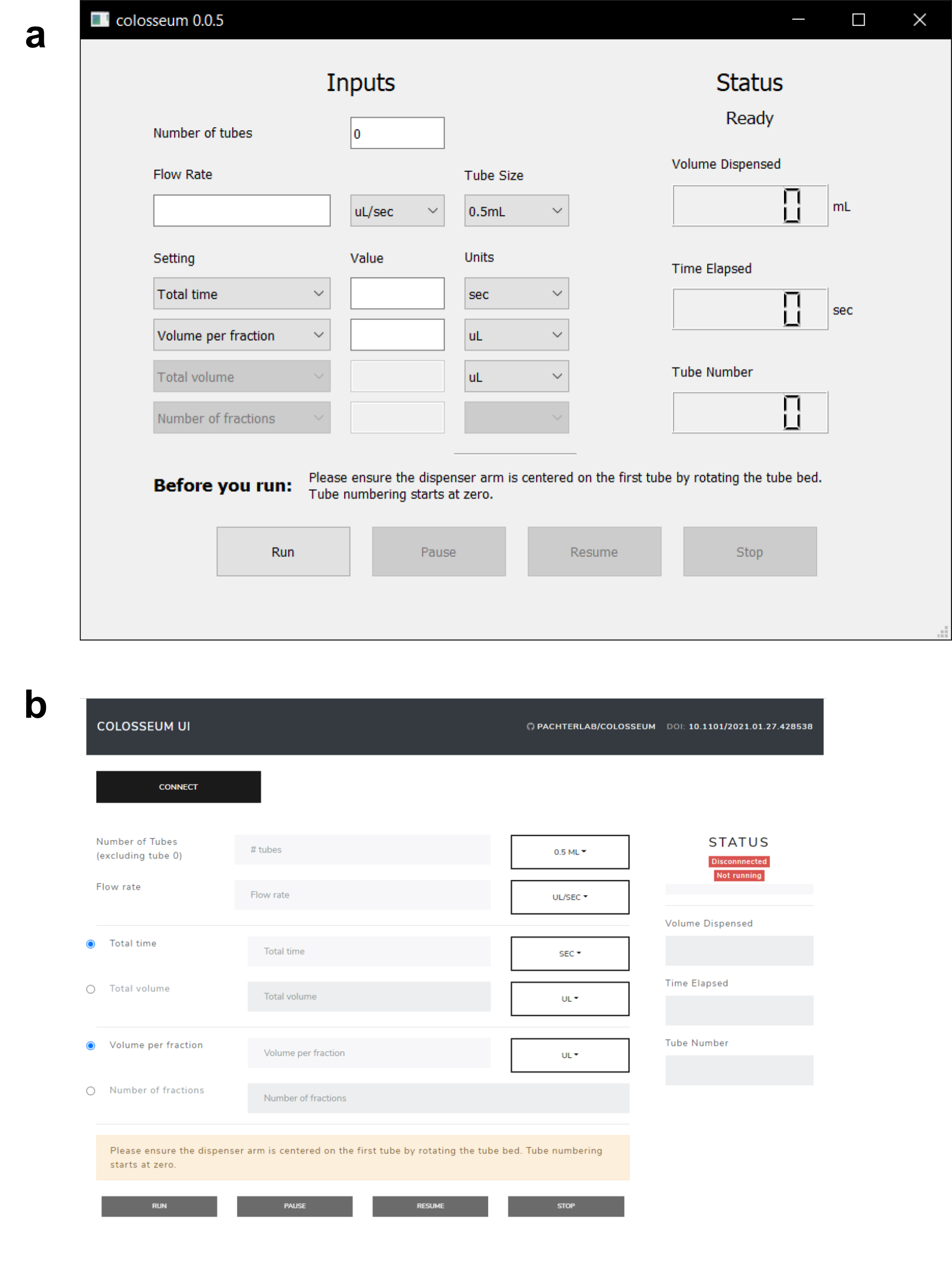


**Supplementary Figure 2:** (a) The Python-based graphical user interface (GUI) and (b) the web-based JavaScript GUI. In both GUI’s left panel displays input boxes for flow rate and collection parameters and the right panel displays experiment progress.


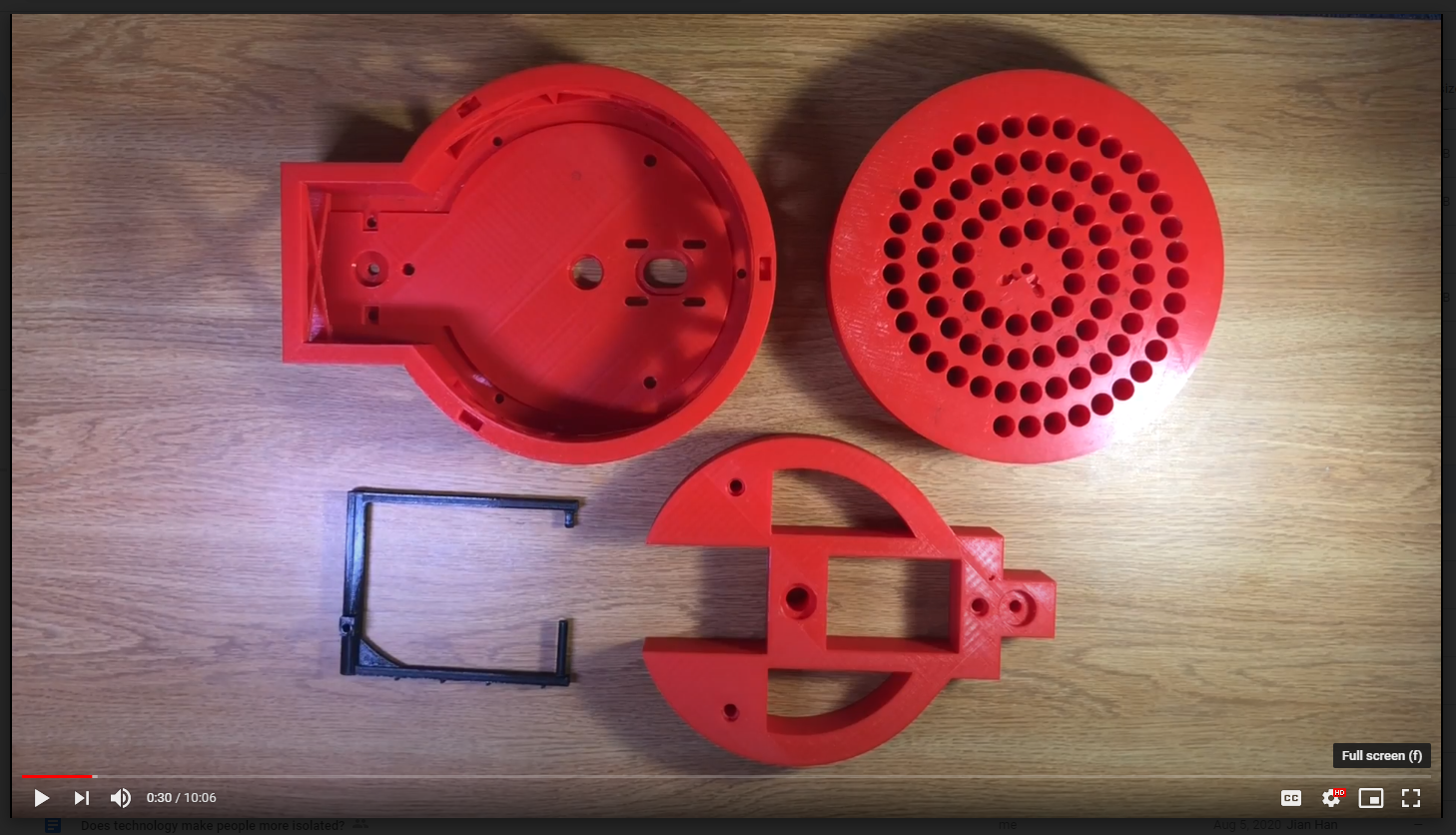


**Supplementary Figure 3:** Assembly video of colosseum. This video guides the user step-by-step through the entire assembly process**.** The video is linked to in the GitHub repository <https://github.com/pachterlab/colosseum>.

| **Model** | **Capacity (# tubes)** | **Price (USD)** |
| --- | --- | --- |
| Cytiva Frac30 [[1]](https://sciwheel.com/work/citation?ids=10365085&pre=&suf=&sa=0) | 30 | 1,615.00 |
| Eldex UFC [[2]](https://sciwheel.com/work/citation?ids=10365088&pre=&suf=&sa=0) | 135 or 160 | 3,971.80 |
| Spectrum Spectra FC [[3]](https://sciwheel.com/work/citation?ids=10365092&pre=&suf=&sa=0) | 174 | 3,393.00 |
| Buchi C-660 [[4]](https://sciwheel.com/work/citation?ids=10365098&pre=&suf=&sa=0) | 12, 30, or 60 | 13,630.11 |
| Open-source | Customizable | <100 |

**Supplementary Table 1:** Costs and capacity of commercial fraction collectors. The costs are based on new, unused models. The capacity of each fraction collector is given by how many tubes the device can hold.

| **Part name** | **Filament weight [length]** | **Print time** | **Supports** |
| --- | --- | --- | --- |
| Tube Rack | 433.80 g [144.281 m] | 31 h 16 min | N |
| Dispenser Arm | 18.53 g [6.162 m] | 1 h 34 min | Y |
| Base | 271.45 g [90.285 m] | 19 h 4 min | Y |
| Base Plate | 174.70 g [58.105 m] | 11 h 26 min | N |
| Total | 898.48 g [298.833 m] | 73 h 30 min |  |

**Supplementary Table 2:** Parts that require 3D printing, including, for each part, the amount of filament (weight and length) required to print, the print time, and whether support is required.

| Parameter 1 | Flow rate | | | |
| --- | --- | --- | --- | --- |
| Parameter 2 | Total time | OR | | Total volume |
| Parameter 3 | Volume per fraction | OR | | Number of fractions |

**Supplementary Table 3:** Table of input parameters for the GUI. The user must input three parameters: flow rate; total time or total volume; and volume per fraction or number of fractions. The user is limited to three parameters to avoid overconstraining the system.

| **Tube #** | **# of 1/4 steps** |
| --- | --- |
| 0 | 84 |
| 1 | 78 |
| 2 | 75 |
| 3 | 70 |
| 4  ... | 64  ... |

**Supplementary Table 4:** The first five rows of the angles between each tube in the tube rack. The angular distances are reported as quarter-steps of the stepper motor. [Data (<https://github.com/pachterlab/BKMGP_2021/blob/main/analysis/archimedian_spiral.ipynb>)]

| Flow rate (mL/hr) | Dwell time (s) |
| --- | --- |
| 720 | 5 |
| 360 | 10 |
| 180 | 20 |
| 90 | 40 |
| 45 | 80 |
| 22.5 | 160 |

**Supplementary Table 5:** Dwell time for each flow rate. To keep the expected fraction volume at 1 mL the flow rate is halved when the dwell time is doubled.

**References**

[Bibliography](https://sciwheel.com/work/bibliography)

[1. Cytiva. Frac30 [Internet]. [cited 27 Jan 2021]. Available: https://web.archive.org/save/https://www.cytivalifesciences.com/en/us/shop/chromatography/tools-and-accessories/fraction-collectors-and-accessories/frac30-p-05647#order](https://sciwheel.com/work/bibliography/10365085)

[2. Amazon. 1243 - UFC Universal Fraction Collector - UFC Universal Fraction Collector, Eldex [Internet]. [cited 27 Jan 2021]. Available: https://web.archive.org/save/https://www.amazon.com/1243-Universal-Fraction-Collector-Eldex/dp/B0731TY8Q8](https://sciwheel.com/work/bibliography/10365088)

[3. Spectrum. Spectra/Chrom® CF-2 Fraction Collector [Internet]. [cited 27 Jan 2021]. Available: https://web.archive.org/save/https://www.spectrumchemical.com/OA_HTML/lab-supplies-products_SpectraChromsup-174sup-CF-2-Fraction-Collector_302400.jsp?section=23230](https://sciwheel.com/work/bibliography/10365092)

[4. VWR. Buchi® Sepacore^TM^ C-660 Fraction Collector [Internet]. [cited 27 Jan 2021]. Available: https://web.archive.org/save/https://us.vwr.com/store/product/4637187/buchi-sepacoretm-c-660-fraction-collector](https://sciwheel.com/work/bibliography/10365098)
