# Supplementary material for: Low-cost, scalable, and automated fluid sampling for fluidics applications
Source: HardwareX. 2021 May 31;10:e00201. doi: 10.1016/j.ohx.2021.e00201 (PMC9123361; doi:10.1016/j.ohx.2021.e00201)
Supplement: Supplementary data 2 [file mmc2.docx]

**Design files**

**Design Files Summary**

| Design file name | File type | Open source license | Location of the file |
| --- | --- | --- | --- |
| colosseum_arm | CAD file | BSD-2 | Available in repository |
| colosseum_base | CAD file | BSD-2 | Available in repository |
| colosseum_baseplate | CAD file | BSD-2 | Available in repository |
| colosseum_tubebed | CAD file | BSD-2 | Available in repository |
